# Comparative Clinical Characteristics of Rheumatic Heart Disease Patients Undergoing Surgical Valve Replacement

**DOI:** 10.7759/cureus.4889

**Published:** 2019-06-12

**Authors:** Hamza I Butt, Ahmad Shahbaz, Haroon Nawaz, Khurram Butt

**Affiliations:** 1 Statistics, Government College University, Lahore, PAK; 2 Cardiac Surgery, Punjab Institute of Cardiology, Lahore, PAK; 3 Internal Medicine, Lahore Medical and Dental College, Lahore, PAK; 4 Internal Medicine, Florida Hospital Orlando, Orlando, USA

**Keywords:** rheumatic heart disease, valve replacement, valve lesions, inflammation, echocardiography, atrial fibrillation

## Abstract

Background

To assess the prevalence patterns of isolated/mixed rheumatic valvular lesions and associated risk factors among rheumatic heart disease (RHD) patients undergoing surgical valve replacement.

Methods

An analytical cross-sectional design was used. Purposive sampling was used to select 87 RHD patients who underwent a first-time valve replacement for mitral, aortic, or both valves between April 1 and October 20, 2016, at Punjab Institute of Cardiology, Lahore, Pakistan. Patients with systemic hypertension, diabetes mellitus type-II, congenital heart defects, coronary artery disease, non-rheumatic valvular degeneration, positive test for hepatitis C, or undergoing concomitant coronary artery bypass graft or a ‘redo’ valve replacement procedure were excluded.

A proforma was used to collect preoperative data on patients’ demographics, laboratory investigations, electrocardiogram (ECG), and transthoracic echocardiography reports.

Results

Age (mean ± S.D.) was 32.79 ± 13.06 years, which was divided into four quartile-based groups. Forty-six (52.9%) cases were males. The majority (56.3%) of patients underwent mitral valve replacement. Mitral regurgitation (MR, 80%) was the most common lesion. Of 71 available ECGs, atrial fibrillation was observed in 46.5% cases. Increasing age group was negatively correlated with MR severity (τ_c_ = -0.188, p-value = 0.033) and positively with aortic stenosis (AS) severity (τ_c_ = 0.141, p-value = 0.010).

No significant elevations were observed for anti-streptolysin O titer, C-reactive protein, and leukocyte count, though the erythrocyte sedimentation rate was abnormally high in 46.94% cases.

Conclusions

MR was the most common lesion. MR was more severe in younger patients whilst AS was more severe in older cases. There is little evidence of ongoing residual inflammation.

## Introduction

Acute rheumatic fever (RF) develops due to an abnormal autoimmune response to group-A beta-hemolytic streptococcal pharyngitis [1], which may affect joints, heart, brain, or subcutaneous tissue and most commonly occurs in children aged five to 15 years [2]. Rheumatic heart disease (RHD) often develops as a complication that may progressively enter a chronic phase characterized by damaged heart valves by recurrent RF episodes. Though successfully eradicated in developed countries, RHD is still the most common global cardiovascular disorder in individuals less than 25 years old [1]. Its continued high prevalence in developing countries translates into high numbers requiring heart valve surgeries, which economically burdens both patients and the respective health ministries [3].

Atrial fibrillation (AF) is a commonly observed cardiac arrhythmia in RHD patients, which increases the risk of thromboembolic stroke [4]. Previous studies have linked the incidence of AF to be positively associated with female gender, the 21-30-year age group [5], and the severity of mitral stenosis (MS) [5-6]. Immunohistological studies have also assessed whether there is ongoing residual inflammation in chronic phase RHD, acting as an additional mechanism for progressive fibrosis and the hemodynamic worsening of heart valves. Commonly studied inflammatory markers include anti-streptolysin-O (ASO) titer [7] and acute phase reactants like C-reactive protein (CRP) [8-10] and erythrocyte sedimentation rate (ESR) [11]. The neutrophil-to-lymphocyte ratio (NLR) is also under study as a novel inflammatory marker linked to higher severity of rheumatic MS [12].

This study aimed at analyzing the clinical characteristics of RHD patients requiring surgical valve replacement. The prevalence patterns of rheumatic valvular lesions and their association with demographic variables and comorbidities were assessed. Ongoing residual inflammation was also assessed through various markers. Cost-intensive perioperative care for heart surgeries means that these findings would be a valuable addition to previous studies in Pakistan, which have not specifically characterized surgical RHD cases.

## Materials and methods

An analytical cross-sectional study design was carried out. Purposive sampling was used to select 87 RHD patients who underwent ‘first-time’ surgical valve replacement between April 1 and October 20, 2016, with bi-leaflet mechanical valves at cardiac surgery units at Punjab Institute of Cardiology (PIC), Lahore, Pakistan. Patients with systemic hypertension, diabetes mellitus type-II, congenital heart defects, coronary artery disease, non-rheumatic valvular degeneration, a positive test for hepatitis C, or undergoing concomitant coronary artery bypass graft or a ‘redo’ valve replacement procedure were excluded. The study was approved by the ethical review committee of the department of research, training, and postgraduate medical education at PIC, Lahore, Pakistan (Ref No. RTPGME-Research-074).

A proforma was designed for data collection on patients’ demographics, preoperative laboratory findings, ECG, and transthoracic echocardiography reports. Informed consent was obtained from each patient. Data were entered into and analyzed using IBM Statistical Package for Social Sciences version 23.0 (IBM Corp., Armonk, NY, US). Bar charts were made using Microsoft Excel (2016 Version; Microsoft Corporation, Washington, United States).

Frequencies and percentages were described for categorical variables, such as gender, procedure (valve replacement) type, surgical outcome, AF status, and bar charts) were used to describe the severity of valvular lesions. Quantitative variables were expressed as mean ±S.D. Age and neutrophil-to-lymphocyte ratio (NLR) were transformed into ordinal categories based on quartiles and tertiles, respectively.

Tests of normality and randomness were carried out before selecting statistical tests for inferential analysis. Pearson’s chi-square test and Fisher’s exact test were used to check the association of AF with age, gender, procedure type, severity of each valvular lesion, and pulmonary hypertension (PHT), respectively. Kendall’s rank correlation (τc) was used to test the correlation of each valvular lesion’s severity with age group, PHT, and NLR, respectively. A cutoff p-value of 0.05 was used for the decision rule.

## Results

The age (mean ±S.D.) of patients was 32.79±13.06 years and they were divided into four age groups based on quartiles. Of the cases, 52.9% were males whilst the majority (87.4%) patients registered in the ‘poor’ procedural category (all medical expenditures covered by the hospital) for the operation. The majority (56.3%) of patients underwent replacement of the mitral valve, followed by the double valve (27.6%) and, finally, the aortic valve (16.1%). Three (3.4%) in-hospital postoperative mortalities were observed, all listing cardiopulmonary arrest as the primary cause (Table 1).

**Table 1:** Demographic and clinical characteristics of patients (n=87) MVR: mitral valve replacement; AVR: aortic valve replacement; DVR: double valve replacement

	Frequency	Percentage (%)
Age Group (Years)		
0-21	23	26.4
22-31	21	24.1
32-41	22	25.3
≥ 42	21	24.1
Age (Mean ±S.D.)	32.79 ± 13.06	
Gender		
Male	46	52.9
Female	41	47.1
Procedural Class		
Poor	76	87.4
Paying	11	12.6
Procedure Type		
MVR	49	56.3
AVR	14	16.1
DVR	24	27.6
Surgical Outcome		
Discharged	84	96.6
In-hospital Mortality	3	3.4

C-reactive protein (CRP) values, available for 64 cases, were elevated in 9 (14.06%) cases, whilst only two (2.82%) out of the 71 available ASO titers were elevated. Leukocytosis was observed in about 10% of patients and around half (46.94%) of the 49 available erythrocyte sedimentation rates (ESR) values were above normal (Table 2).

**Table 2:** Inflammatory markers ASO: anti-streptolysin-O; CRP: C-reactive protein; NLR: neutrophil-to-lymphocyte ratio

	Frequency	Percentage (%)
ASO Titer (IU/ml)		
<200	69	97.18
≥200	2	2.82
CRP (mg/l)		
<6	55	85.94
≥6	9	14.06
ESR (mm 1st hour)		
1-20	26	53.06
>20	23	46.94
NLR Groups		
NLR≤1.66	29	33.33
1.66 < NLR≤2.12	29	33.33
NLR>2.12	29	33.33

Comparison between two-dimensional echocardiographic measurements reveals differences between the ‘procedure-type’ categories. Left atrium (LA) size is significantly higher in the two operation types involving the mitral valve (p-value = 0.000), whilst left ventricle (LV) internal dimensions (diastolic and systolic) and LV mass were significantly higher for the two operation types involving the aortic valve (p-values = 0.005, 0.001, and 0.000, respectively) (Table 3).

**Table 3:** B-M mode echocardiographic dimensions classified by valve replacement type SMC denotes significant multiple comparisons, namely, MA=MVR vs. AVR, MD=MVR vs.DVR, and AD=AVR vs. DVR, respectively. MVR: mitral valve replacement; AVR: aortic valve replacement; DVR: double valve replacement; LVISD: left ventricular internal systolic diameter; LVPWD: left ventricular posterior wall dimensions; LVIDD: left ventricular internal dimension-diastole; LVIDS: left ventricular internal dimension in systole; EF: ejection fractio

	MVR (n=45)	AVR (n=14)	DVR (n=23)	Kruskal Wallis H-Test (2 d.f)		*SMC
				H	p-value	
LA (mm)	55.22 ± 1.60	38.64 ± 1.25	52.17 ± 2.01	26.09	0.000	MA, AD
LVISD (mm)	9.84±0.25	12.36±0.54	10.74±0.37	14.43	0.001	MA
LVPWD (mm)	9.69±0.22	12.36±0.55	10.61±0.33	16.06	0.000	MA
LVIDD (mm)	52.31±1.19	60.57±3.35	58.09±2.51	10.51	0.005	MA, MD
LVIDS (mm)	33.56±1.04	40.00±2.39	40.74±2.14	13.31	0.001	MA, MD
EF (%)	59.23±0.77	53.29±2.52	57.04±1.18	4.93	0.085	-
LV Mass (g)	197.76±9.11	343.44±30.60	267.96±19.16	25.33	0.000	MA, MD

**Figure 1:**
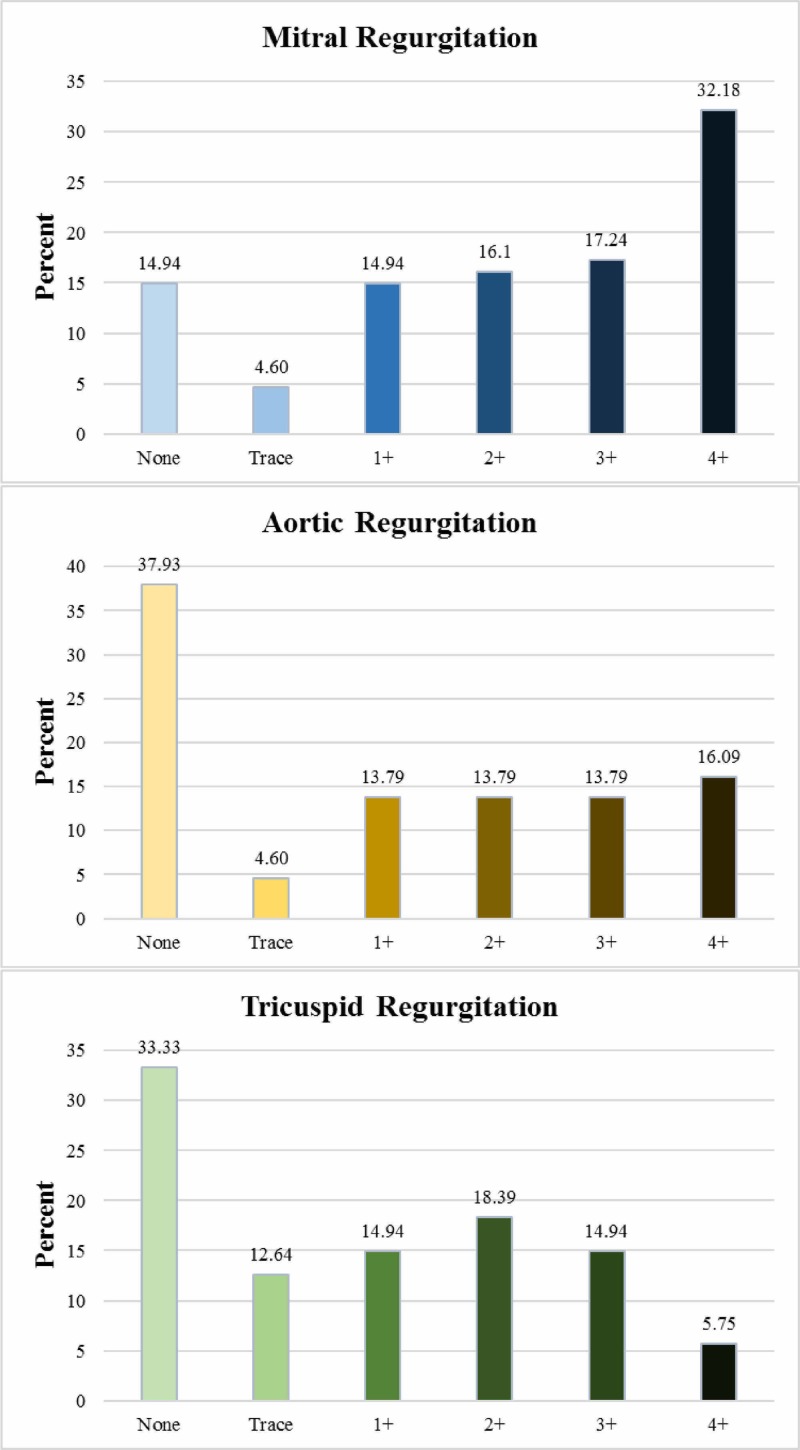
Valvular regurgitant lesions (MR, AR, TR) MR: mitral regurgitation; AR: aortic regurgitation; TR: tricuspid regurgitation

**Figure 2:**
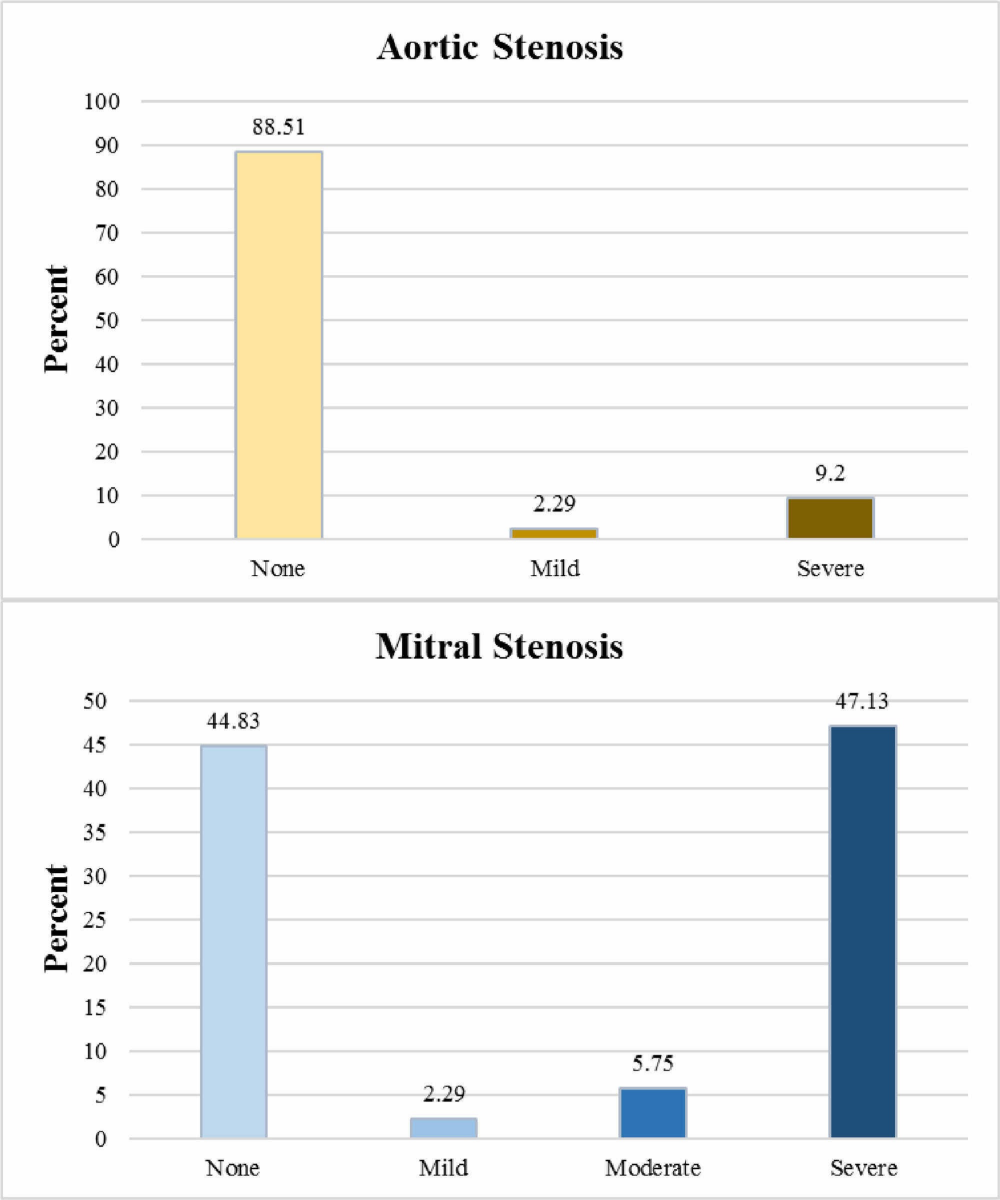
Valvular stenotic lesions (AS, MS) AS: aortic stenosis; MS: mitral stenosis

Valvular lesion severities were graded according to semi-quantitative criteria with reference to the American Society of Echocardiography’s recommendations [13-14]. Mitral regurgitation (MR) was the most common regurgitant lesion, with about 80% of cases having a ‘grade 1+’ or higher level. Aortic regurgitation (AR) was the second most common, with 57.5%, followed by tricuspid regurgitation (TR), with 54% of cases having a ≥ grade 1+ level. MS was the most common stenotic lesion, being present in 55.17% of cases; aortic stenosis (AS) was observed in about 11.5% cases and tricuspid stenosis wasn’t observed in any case (Figures 1-2).

The bivariate tests of association listed in Table 4 showed that the presence of AF was significantly associated with age group (p-value = 0.022), procedure type (p-value = 0.013), TR severity (p-value = 0.028), and AS severity (p-value = 0.017). Subsequent analyses of cross-tabulations also showed that AF was more common in higher age groups, most common in MVR cases, showed an increasing trend with TR, and a decreasing trend with AS severity.

**Table 4:** Tests of association *significant at α=0.05 AF: atrial fibrillation; MR: mitral regurgitation; AR: aortic regurgitation; TR: tricuspid regurgitation; AS: aortic stenosis; MS: mitral stenosis; PHT: pulmonary hypertension

	Pearson’s χ^2^ test					
AF	Age group		Gender		Procedure Type	
	9.66 (0.022*)		0.17 (0.676)		8.67 (0.013*)	
	Fisher’s exact test					
	MR	AR	TR	MS	AS	PHT
	8.73 (0.107)	6.89 (0.227)	11.73 (0.028*)	2.21 (0.587)	7.38 (0.017*)	8.28 (0.075)

Table 5 shows tests of Kendall’s rank correlation carried out to check the directional association of valvular lesion severity with age group, PHT level, and NLR group, respectively. Increasing age group showed a significant negative correlation with MR severity (τc = -0.188, p-value = 0.033) and a positive correlation with AS severity (τc = 0.141, p-value = 0.010). PHT showed a significant positive correlation with MR, TR, and MS and a negative correlation with AR severity. NLR group was not correlated with any of the lesions.

**Table 5:** Tests of Kendall’s rank correlation of valvular lesion severity with age group, PHT, and NLR, respectively *significant at α=0.05 MR: mitral regurgitation; AR: aortic regurgitation; TR: tricuspid regurgitation; MS: mitral stenosis; TS: tricuspid stenosis

	Age Group	PHT	NLR
MR	-0.19 (0.033*)	0.27 (0.001*)	0.03 (0.735)
AR	-0.17 (0.058)	-0.23 (0.009*)	-0.11 (0.272)
TR	-0.01 (0.885)	0.43 (0.000*)	0.16 (0.112)
MS	0.13 (0.106)	0.32 (0.000*)	0.14 (0.138)
TS	0.14 (0.010*)	-0.09 (0.075)	0.06 (0.177)

## Discussion

In developing countries, rheumatic fever and carditis still constitute a major public health problem. This is a disease of populations that have difficult access to health care. These patients have special characteristics that differ from those we generally see in economically developed countries. They are usually young, poor, uneducated, and have low compliance with prophylaxis/therapy. In addition, they usually experience great difficulty in accessing medical care. By contrast, rheumatic fever has mostly disappeared in developed countries, except in those with large immigrant contingents because medical care is usually easily accessible. In these regions, we now rarely see new cases of acute rheumatic carditis, but older patients, who had the acute form of the disease some decades ago, still present with sequelae of rheumatic disease, in their fifties and sixties, requiring valve surgery [15].

Rheumatic valvular disease continues to be a significant disease entity in countries like Pakistan. The National Institute of Cardiovascular Disease reported in 2004 that 8%-29% of hospital admissions and 62% of the surgical load in tertiary care cardiovascular centers in Pakistan is attributable to RF/RHD; furthermore, only 8% of established RF cases were on prophylactic medication, putting them at risk of recurrent RF episodes [16]. Previous studies in the country have described the prevalence patterns of RHD and rheumatic valvular lesions in hospital- or community-based settings. An RHD prevalence of 5.7 per 1000 was reported in 2004 for a rural setting in Rahim Yar Khan [17]. A study in Lahore reported a prevalence of 21.9 per 1000 in urban/semi-urban schoolchildren [18], which is much higher than a pooled prevalence of 12.9 per 1000 for 37 countries reported in 2014 [19]. A screening study on valvular lesions in 100 RHD patients at two teaching hospitals of Hyderabad, Sindh, reported mitral stenosis (MS, 48%) as the most common, followed by mitral regurgitation (MR, 42%) [20]. Similar patterns were reported in an echocardiographic screening of 3060 RHD patients at Lady Reading Hospital, Peshawar, with MS present in 70% followed by MR in 58.59% cases [21]. A large-scale retrospective screening of RHD patients admitted at Punjab Institute of Cardiology, Lahore, between 2004 and 2008, however, reported MR (56%) as most common followed by tricuspid regurgitation (TR) and aortic regurgitation (AR); furthermore, regurgitant lesions were more severe in younger patients [22].

Valvular lesion patterns in our findings are similar to those of previous studies in Hyderabad and Peshawar, which reported mitral valve lesions as most common, followed by aortic valve lesions in RHD patients [20-21], though these studies reported MS and not MR as the most prevalent lesion. A previous study at PIC did report MR as the most prevalent [22], suggesting that there may be geographical location-based variations in these prevalence patterns and could be linked to access to health care.

The order of rheumatic valve involvement in our study was similar to findings in nearby Asian countries. A study in India indicated mitral valve disease as most common at 60.2%, followed by aortic and tricuspid valvular diseases subsequently [23]. A recent study amongst 235 RHD patients at a tertiary care facility in central Nepal also reported isolated mitral valve involvement (46.8%) as most common, followed by mixed mitral/aortic (33.62%) and isolated aortic (9.36%) valve, respectively [24].

The AF-AS relationship observed in our study contrasts the strongly positive association reported by a previous study in Sweden [25]; furthermore, the higher incidence of AF in females (72.72% vs 27.27%) [5] and the strong association of rheumatic MS with AF reported in several studies [5-6,26] was not observed in our study.

We also evaluated the likelihood of residual inflammation in valve replacement candidates via certain inflammatory markers. The usefulness of NLR as an inflammatory marker could not be established in our findings. A previous study showed a positive association of NLR with the severity of rheumatic MS [27]; furthermore, a case-control study in Egypt also reported a higher mean NLR count in severe multi-valvular RHD cases as compared to isolated MR, isolated MS, and mixed MR/MS patients [28]. Furthermore, significant elevations of CRP in chronic RHD cases reported previously [9,27] were difficult to observe in our study since CRP levels were only available as categories (<6 or ≥6 mg/l).

This study was subject to some limitations. Measurements for inflammatory markers and preoperative ECG were not available for all patients. The presence or absence of AF was assessed on single preoperative ECG, which could fail to detect cases of paroxysmal AF. Furthermore, immunohistochemistry assays more comprehensively determine the role of inflammatory markers as compared to observational findings in our study, e.g., a recent study in China indicated the role of M1 macrophages in progressing atrial inflammation and thrombus formation in rheumatic MS patients with AF [29]. Finally, the small sample size hindered the observation of gender-based differences, such as the varied aortic valve pathology observed in a previous study [30].

## Conclusions

This study demonstrates that among RHD valve replacement patients, MR was more severe in younger patients whilst AS was more severe in older cases. The observed relationship of AF with MS and AS and gender differed from the findings of previous studies. Measurements for inflammatory markers indicate a lack of concrete evidence of ongoing residual inflammation in chronic phase RHD.
